# Profils de résistance aux médicaments antituberculeux chez les patients atteints de tuberculose pulmonaire résistante à la rifampicine à Yaoundé, Cameroun

**DOI:** 10.48327/mtsi.v6i2.2026.865

**Published:** 2026-06-15

**Authors:** Alain KUABAN, Jurgen NOESKE, Christopher KUABAN

**Affiliations:** 1 Département de médecine interne et spécialités, Faculté de médecine et des sciences biomédicales, Université de Yaoundé, Cameroun; 2 Service de pneumologie, Hôpital Jamot de Yaoundé, Cameroun; 3 Consultant indépendant, Yaoundé, Cameroun; 4 Faculté des sciences de la santé, Université de Bamenda, Cameroun

**Keywords:** Tuberculose, Antituberculeux, Résistance, Rifampicine, Cameroun, Afrique subsaharienne, Tuberculosis, Antituberculosis drug, Resistance, Rifampicin, Cameroon, Sub-Saharan Africa

## Abstract

**Introduction:**

Le dépistage de la tuberculose pulmonaire multirésistante (TBP-MR) en utilisant la résistance à la rifampicine obtenue par le test Xpert MTB/RIF comme marqueur de substitution de cette forme de tuberculose risque de conduire à un traitement sous-optimal si sa résistance aux autres médicaments du régime thérapeutique n’est pas déterminée. L’objectif de cette étude était d’analyser les profils de résistance aux médicaments antituberculeux chez les patients atteints de la tuberculose pulmonaire résistante à la rifampicine (TBP-RR) traités par le régime thérapeutique standardisé de 9 à 11 mois à Yaoundé, Cameroun.

**Matériel et méthode:**

Il s’agissait d’une étude transversale rétrospective des résultats des tests de sensibilité aux médicaments antituberculeux de patients atteints de TBP-RR traités par le régime thérapeutique standardisé de 9 à 11 mois au centre spécialisé de traitement de la TBP-MR de l’Hôpital Jamot de Yaoundé entre 2013 et 2022.

**Résultats:**

Au total, 322 patients atteints de TBP-RR ont été inclus dans l‘étude. Parmi eux, 281 (87,3 %) étaient résistants à au moins un des médicaments testés. Le profil de résistance était de 43,2 % pour un seul médicament, 41,6 % pour deux médicaments et 2,5 % pour trois médicaments ou plus. La résistance à l'isoniazide était la plus fréquente (87,3 %), suivie de l‘éthambutol (42,2 %), des fluoroquinolones (3,4 %) et de l'amikacine (1,9 %).

**Discussion - Conclusion:**

La majorité des patients TBP-RR à Yaoundé avait une TBP-MR, et la TB pré-ultrarésistante était rare. Malgré cela, la stratégie de recherche de cas de résistance aux médicaments basée sur GeneXpert (qui identifie uniquement celle à la rifampicine) peut conduire à l’initiation d’un traitement sous-optimal pour une proportion non négligeable de patients dans notre milieu. Cela pourrait conduire à une amplification de la résistance et à la transmission de souches avec une résistance de plus en plus avancée au sein de la communauté. Nous recommandons que le Programme national de lutte contre la tuberculose (PNLT) du Cameroun adopte et généralise rapidement le nouveau régime thérapeutique court recommandé par l’Organisation mondiale de la Santé, compte tenu de la non-disponibilité des tests rapides dans notre pays permettant de détecter la résistance aux différents médicaments utilisés dans le régime standardisé de 9 à 11 mois.

## Abstract (en)

**Introduction:**

Screening for multidrug-resistant pulmonary tuberculosis (MDR-PTB) by using the Xpert MTB/ RIF test to detect rifampicin resistance as a surrogate marker for this form of tuberculosis may lead to suboptimal treatment if resistance to other drugs in the treatment regimen is not also determined. The objective of this study was to analyze the patterns of drug resistance to anti-tuberculosis drugs in patients with rifampicin-resistant pulmonary tuberculosis (RR-PTB) who were treated with the standardized 9-11-month regimen in Yaoundé, Cameroon.

**Materials and methods:**

This retrospective, cross-sectional study examined the results of anti-tuberculosis drug susceptibility tests in RR-PTB patients who received the standardized 9-11-month treatment regimen at the specialized MDR-PTB treatment center of the Jamot Hospital in Yaoundé from 2013 to 2022.

**Results:**

A total of 322 RR-PTB patients were included in the study. Of these, 281 (87.3%) were resistant to at least one of the tested drugs. The pattern of resistance was 43.2% for one drug, 41.6% for two drugs, and 2.5% for three or more drugs. Resistance to isoniazid was the most common (87.3%), followed by ethambutol (42.2%), fluoroquinolones (3.4%), and amikacin (1.9%).

**Discussion – Conclusion:**

The majority of RR-PTB patients in Yaoundé had MDR-PTB, and pre-extensively drug-resistant TB was rare. However, the GeneXpert-based strategy for identifying drug-resistant cases (which detects only rifampicin resistance) could lead to the initiation of suboptimal treatment for a non-negligible proportion of patients in our setting. This could result in the amplification of resistance and the transmission of increasingly resistant strains within the community. We recommend that the National Tuberculosis Control Program (NTCP) of Cameroon should adopt and rapidly roll out the new short-course treatment regimen recommended by the World Health Organization given the lack of rapid tests in our country capable of detecting resistance to the various drugs used in the standard 9-11-month regimen.

**Figure:**
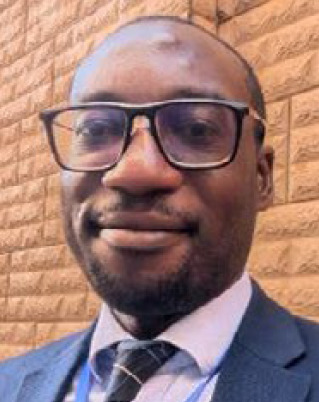

## Introduction

La tuberculose pulmonaire (PTB) est la première cause de décès due à un agent infectieux unique dans le monde, se classant au-dessus de l’infection du virus de l’immunodéficience humaine/ syndrome d’immunodéficience acquise (VIH/ sida) [13].

Malgré les efforts considérables déployés, son contrôle est en partie entravé par la TBP résistante aux médicaments, en particulier sa forme multirésistante (TBP-MR). Cette forme est définie comme une TBP résistante à, au moins, la rifampicine (R) et l’isoniazide (H). Ces deux médicaments sont les moins chers, les mieux tolérés et les plus efficaces pour le traitement de la TBP sensible aux médicaments [15].

Parallèlement, il a été démontré que la grande majorité des patients porteurs de souches de TBP résistantes à la R (TBP-RR) ont un pronostic aussi défavorable que les cas de TBP-MR lorsqu’ils sont traités uniquement avec des antituberculeux de première ligne [4,8]. Pour cette raison, les patients atteints de TBP-RR sont désormais traités comme les cas de TBP-MR.

La disponibilité au Cameroun du test Xpert MTB/RIF (Cepheid Sunnyvale, CA, USA), qui détecte non seulement *Mycobacterium tuberculosis* dans les échantillons mais aussi sa résistance à la rifampicine, permet de dépister rapidement les cas suspects de TBP ayant une résistance à la rifampicine et de les traiter immédiatement avec le régime thérapeutique de la TBP-MR si cela est indiqué. Ce régime comprend des antituberculeux de première et de deuxième ligne.

Bien que cela puisse être considéré comme un progrès important, il convient de reconnaître que l’utilisation du seul test Xpert MTB/RIF pour identifier les patients à traiter par le régime standardisé court de 9 à 11 mois de la TBP-MR, peut contribuer à des pratiques de traitement sous-optimales. Cela peut résulter du fait que ce traitement n’est pas entièrement guidé par des résultats confirmés par des tests de sensibilité aux médicaments (TSM) en laboratoire pour tous les médicaments inclus. L’absence de résultats de TSM pour ces patients au début du traitement peut donc conduire à un risque de résistance non identifiée aux médicaments, à ceux de première ligne, mais aussi de deuxième ligne. D’autant plus qu’il est aujourd’hui signalé qu’une résistance aux médicaments nouveaux et réutilisés est en train d’émerger [9,10,11,12].

La connaissance des profils de résistance aux médicaments chez les patients atteints de TBP-RR dans notre contexte est donc essentielle pour guider leur prise en charge et réduire le risque de résistance supplémentaire.

L’objectif de cette étude était d’analyser les profils de résistance aux médicaments antituberculeux observés chez les patients atteints de TBP-RR recevant le régime de traitement standardisé de 9 à 11 mois contre la TBP-MR à Yaoundé, au Cameroun.

## Matériel et méthode

L’étude a été menée dans le centre spécialisé de traitement de la TBP-MR de l’Hôpital Jamot de Yaoundé (HJY), seule structure de référence pour Yaoundé et ses environs.

Dans ce centre, conformément aux directives du Programme national de lutte contre la tuberculose (PNLT) du Cameroun [5] et des recommandations de l’Organisation mondiale de la Santé (OMS) [15], tous les patients atteints de tuberculose pulmonaire (TBP) antérieurement traités, ainsi que les contacts de patients atteints de tuberculose multirésistante/résistantes à la R (TBP-MR/RR) présentant des symptômes évocateurs de TBP, soumettent deux échantillons de crachats sur deux jours consécutifs au Laboratoire national de référence de la tuberculose du Centre Pasteur du Cameroun à Yaoundé pour examen microscopique à la recherche de bacilles acido-alcoolo-résistants.

Les échantillons sont également examinés par le test Xpert MTB/RIF pour évaluer s’ils contiennent des souches de *M. tuberculosis* résistantes ou non à la rifampicine. Les résultats des patients sont immédiatement communiqués au centre spécialisé pour une prise en charge thérapeutique appropriée. Le reste des crachats reçus au laboratoire de référence pour chacun des patients présentant une résistance à la rifampicine au test Xpert MTB/ RIF est mis en culture en milieu liquide.

La culture et les tests de sensibilité aux médicaments antituberculeux sur milieux liquides ont été réalisés à l’aide de BD BACTEC MGIT 960 (BD Diagnostics; Sparks USA). Les tubes BACTEC MGIT de 7 ml avec le kit de supplémentation MGIT, ainsi que les flacons combinés PANTA et OADC ont été utilisés pour la culture, et le kit BACTEC MGIT SIRE pour le test de sensibilité aux antituberculeux de première ligne.

Un TSM de première ligne et de certains médicaments de deuxième ligne des souches de *M. tuberculosis* isolées des cultures a été réalisé à l’aide du test par hybridation sur bandelettes (LPA) Génotype MTBDR plus version 2 (Bruker; Nehren, Allemagne) et un des tests conventionnels de sensibilité phénotypique aux médicaments par la méthode de proportion indirecte, décrite par Canetti *et al*. [6], et/ou du *Mycobacteria Growth Indicator Tube* (BD BACTEC MIGIT 960).

Les concentrations critiques de l’OMS, telles que recommandées dans le Module 3 de son manuel opérationnel sur la tuberculose [16], ont été utilisées pour les tests de sensibilité aux antituberculeux.

Les médicaments actuellement testés dans le laboratoire de référence comprennent des médicaments de première ligne : R, H et éthambutol (E); ils comprennent aussi des médicaments de deuxième ligne : quinolones (ofloxacine, moxifloxacine (Mfx)) et aminosides (amikacine, kanamycine). Les résultats de la culture et du TSM sont communiqués au centre de traitement spécialisé où ils sont enregistrés dans le registre de traitement de la TBP-MR et sur la fiche de traitement de chaque patient.

Jusqu’en 2022, tous les patients atteints de TBP-MR/RR étaient traités par un traitement standardisé de 9 à 11 mois, comprenant une phase intensive de 4 à 6 mois avec administration quotidienne de : Mfx, amikacine (Am), prothionamide (Pto), clofazimine (Cfz), H à haute dose (Hh), E et pyrazinamide (Z) [4-6Am-Mfx-Pto-Cfz-Hh-E-Z]. Cette phase était suivie d’une phase supplémentaire de cinq mois comprenant Mfx-Cfz-E-Z, également administrée quotidiennement. En 2023, le régime a été modifié par le remplacement de l’Am par la bédaquiline (Bdq), administrée pendant les 6 premiers mois du traitement (4-6 Bdq (6)-Mfx-Cfz-Z-E-Hh-Pto/5 Mfx-Cfz-Z-E). L’étude était transversale et rétrospective, incluant tous les patients de 15 ans et plus, atteints de TBP-RR et mis sous traitement au centre spécialisé de traitement de la TBP-MR de l’HJY de janvier 2013 à décembre 2022. Les patients dont les dossiers étaient incomplets ont été exclus de l’étude. Ceux dont les tests Xpert MTB/RIF avaient montré une souche de *M. tuberculosis* résistante à la rifampicine, mais dont les cultures n'avaient révélé aucune croissance ou avaient été contaminées, ont également été exclus.

Tous les patients éligibles atteints de TBP-RR ayant été mis sous traitement pendant la période d’étude ont été identifiés à l’aide du registre de traitement de la TBP-MR et de leurs fiches de traitement. Pour chaque patient, les informations suivantes ont été extraites de ces documents et enregistrées sur une fiche de collecte de données préétablie : numéro d’enregistrement, âge, sexe, catégorie du patient selon son antécédent de traitement antituberculeux antérieur (rechute, échec thérapeutique, échec de retraitement, retour après perte de suivi, contact avec un cas connu de TBP-MR/RR) et statut VIH.

Les résultats du test de sensibilité aux différents antituberculeux de chaque patient inclus ont également été extraits et enregistrés. Le profil de résistance de ces patients a été en outre catégorisé selon les définitions suivantes [15]:

- patient atteint de TBP-MR : défini comme un patient dont l'isolat de crachat en culture était positif pour *M. tuberculosis* et dont la résistance *in vitro* à au moins la H et la R a été établie;
- patient atteint de tuberculose pré-ultrarésistante aux médicaments (TB pré-UR) : défini comme un patient ayant une souche de TBP-MR résistante aux fluoroquinolones (ofloxacine ou Mfx);
- patient atteint de tuberculose ultra-résistante aux médicaments (TB-UR) : défini comme un patient hébergeant des souches qui satisfont à la définition de la TBP-MR/RR, et qui sont également résistantes aux fluoroquinolones et à au moins un médicament supplémentaire du groupe A.

Les données ont été saisies en double sur ordinateur à l’aide du logiciel *EpiData Entry Client* version 4.6.0.6. Les discordances ont été identifiées et résolues par vérification du dossier papier original. L’analyse des données a été réalisée à l’aide du logiciel *EpiData Analysis* version 3.3.0. Des analyses descriptives ont été effectuées pour les variables démographiques, cliniques et de laboratoire, et présentées sous forme de moyenne avec écart-type (ET), de nombres et/ou pourcentages. L’étude a été approuvée par le Comité d’éthique institutionnel (CEI) de la Faculté de médecine et des sciences biomédicales de l’université de Yaoundé 1 (Réf N^o^ : 1264/UY1/FMSB/VDRC/ DAASR/CSD). S’agissant d’une étude rétrospective, le consentement éclairé des participants n'a pas pu être obtenu. Les informations des patients ont été anonymisées et dépersonnalisées avant l'analyse. L'autorisation administrative pour mener l‘étude au Centre spécialisé de traitement de la tuberculose multirésistante de l'HJY a été obtenue de l'administration de l’hôpital (Réf. N^o^ : 444/L/MINSANTE/SG/DHJY/mm). L‘étude a été menée conformément à la Déclaration d'Helsinki révisée en 2013.

## Résultats

Un total de 351 patients atteints de TBP-RR a été mis sous traitement au centre spécialisé de la TBP-MR de l’HJY au cours de la période d’étude. Parmi eux, 29 (8,3 %) ont été exclus de l’étude en raison de dossiers incomplets, incluant l’absence de résultats de tests de sensibilité aux médicaments due à l’absence de croissance de *M. tuberculosis* en culture ou à des cultures contaminées. Cependant, aucune différence statistique significative n’a été observée en ce qui concerne l’âge, le sexe et le statut VIH entre les patients exclus et les 322 patients retenus dans l’étude.

Sur ces 322 patients, on dénombrait 198 (61,5 %) hommes et 124 (38,5 %) femmes, avec un âge moyen de 36,2 ± 12,7 ans (intervalle : 15-81 ans). Cent un d’entre eux (31,4 %) étaient séropositifs au VIH, et la majorité (258, soit 80,1 %) avait déjà antérieurement été traitée pour la tuberculose. Le Tableau I résume les caractéristiques générales des 322 patients inclus dans l’étude.

**Tableau I:** Caractéristiques générales de la population d’étude

Caractéristiques	Nombre (N = 322)	%
Âge (en années)				
moyenne ± écart type	36,2 ± 12,7	
intervalle	15-81	
Sexe				
homme	198	61,5
femme	124	38,5
Statut VIH				
positif	101	31,4
négatif	221	68,6
Catégorie de traitement anti-TBP antérieur				
échec du traitement de première ligne (TPL)	121	37,6
rechute après TPL pour les nouveaux cas	110	34,2
retour après abandon du TPL pour les nouveaux cas	27	8,4
nouveau cas de TBP en contact avec un cas connu de TBP-MR/RR	49	15,2
autres	15	4,7

Parmi les 322 patients atteints de TBP-RR étudiés, 41 (12,7 %) étaient sensibles à tous les antituberculeux testés, à l’exception de la R. La résistance à un ou plusieurs médicaments a été observée chez 281 patients (87,3 %). Celle à la H était relevée chez la totalité des 281 (100 %) patients TBP-RR présentant une résistance supplémentaire, suivie par l’E chez 136 (48,4 %) de ces patients. La résistance à la H seule a été observée chez 139 (43,2 %) des 322 patients TBP-RR inclus et était associée à une résistance à d’autres médicaments testés chez les 183 cas restants (56,8 %). Les profils des résistances aux antituberculeux testés sont présentés dans le Tableau II.

**Tableau II:** Profils de résistance aux autres antituberculeux chez 281 patients atteints de TBP-RR à Yaoundé, Cameroun

Résistant au(x) médicament(s)	Population d'étude (n)	% de la population résistante (n = 281)
Un médicament		
H	139	43,2
Deux médicaments		
HE	128	39,6
HFq	5	1,6
HKm	1	0,3
Total	134	41,6
Trois médicaments		
HEFq	3	0,9
HEKm	2	0,6
Total	5	1,6
Quatre médicaments		
HEKmFq	3	0,9
Au moins un médicament	281	87,3

H = isoniazide; E = éthambutol; Fq = fluoroquinolones; Km = kanamycine

Sur les 281 patients TBP-RR ayant une résistance à au moins un médicament, la résistance à un seul médicament était observée chez 139 (49,6 %) d’entre eux, à deux médicaments chez 134 (47,6 %) et à trois médicaments ou plus chez 8 (2,9 %). La résistance à la H était toujours présente lorsqu’il y avait une résistance à un autre médicament.

Le Tableau III présente la répartition des 322 patients atteints de TBP-RR présentant une résistance à au moins un des médicaments testés, selon leur classe de profils de résistance.

**Tableau III:** Répartition de 322 patients atteints de TBP-RR par classe de profils de résistance aux antituberculeux

Profil de résistance aux médicaments	Patients TBP-RR (n = 322)	%
Résistance à la R uniquement	41	12,7
Multirésistance aux médicaments	270	83,9
Pré-ultrarésistance aux médicaments	11	3,4
**Total**	**322**	**100,0**

La proportion de patients présentant uniquement des souches de TBP-MR était de 83,9 % alors que celle de TBP pré-UR n’était que de 3,4 %.

## Discussion

À notre connaissance, cette étude est la première à examiner les profils de résistance aux antituberculeux de première et de deuxième lignes chez des patients atteints de tuberculose résistante à la R au Cameroun.

Chez ces patients, le taux global de résistance à au moins un des antituberculeux testés dans cette étude était de 87,3 %. Ce taux concorde avec des résultats similaires observés en Sierra Leone et en Ouganda [1,2]. Ce taux global élevé de résistance aux médicaments pourrait s’expliquer par le fait que la plupart des patients atteints de TBP-RR inclus dans notre étude (84,5 %) avait des antécédents de traitement antituberculeux antérieur. Le nombre de cas résistants à la seule R était de 41. La monorésistance à la R est très rare et ces cas justifieraient une analyse complémentaire des dossiers cliniques de ces patients.

La majorité des patients atteints de TBP-RR (281, soit 87,3 %) était résistante à au moins un des médicaments testés, la H étant le plus fréquemment concernée, suivie de l’E (48,4 %). Les taux élevés de résistance à la H et à l’E observés chez les patients atteints de TBP-RR dans cette étude remettent en question la pertinence de leur inclusion dans le régime entièrement oral de 9 à 11 mois pour les personnes atteintes de TBP-MR/RR, régime encore utilisé au Cameroun pour une grande partie de ces patients. Il est désormais établi que des mutations au niveau du promoteur *inhA* ou des mutations dans les régions *inhA* et *KatG* de *M. tuberculosis* confèrent une résistance à la H. La résistance est considérée de faible niveau lorsque seules des mutations *inhA* sont présentes, et de haut niveau lorsque des mutations dans le gène *KatG* seul ou des mutations combinées dans les régions du promoteur *inhA* et de *KatG* sont observées. Il est également connu que les mutations au niveau du promoteur *inhA* sont associées à une résistance à l’éthionamide et à la prothionamide. Cette dernière est incluse dans le régime entièrement oral de 9 mois. Actuellement au Cameroun, ces mutations ne peuvent pas être déterminées.

En conséquence, la présence de mutations du promoteur *inhA* et de *KatG* suggère que la H à forte dose et les thionamides pourraient ne pas être efficaces, et que le régime entièrement oral de 9 à 11 mois pourrait ne pas être utile pour tous les patients atteints de TBP-MR/RR [15]. Il a été proposé cependant qu’en l’absence d’informations sur les profils de mutation pour un patient donné dans des pays comme le Cameroun, la décision de traiter un malade avec ce régime peut être éclairée par la fréquence de l’occurrence concomitante des deux mutations obtenues à partir de la surveillance de la résistance aux médicaments [14]. Malheureusement, notre pays, comme de nombreux pays à revenu faible et intermédiaire, ne dispose pas actuellement de tels systèmes de surveillance permettant d’avoir les informations sur ces mutations.

La résistance aux deux médicaments de deuxième ligne testés dans cette étude était faible. Une résistance aux fluoroquinolones a été observée chez 11 (3,9 %) des 281 patients atteints de TBP-RR présentant une résistance à au moins un des médicaments testés. Ce taux est inférieur au taux de 7 % rapporté par Rao *et al*. [17] au Pakistan. Comme aucune donnée n’est disponible à ce jour concernant la sensibilité de la TBP-RR aux quinolones au Cameroun, ce résultat revêt une grande importance. En effet, le taux de résistance aux quinolones observé dans cette étude, bien que faible, devrait être considéré comme un signal d’alarme pour le traitement de ce groupe de patients atteints de tuberculose, car il peut conduire à des cas de pré-ultra-résistance aux médicaments. La situation actuelle pourrait éventuellement s’aggraver car les quinolones ont un potentiel au Cameroun pour être utilisés à la fois sur une base auto-administrée et sur ordonnance dans le traitement de maladies infectieuses autres que la tuberculose. Les mises en garde contre l’utilisation abusive de ce médicament majeur de deuxième ligne sont d’une importance capitale, car des taux plus élevés de résistance aux quinolones menaceraient l’efficacité des régimes entièrement oraux plus courts recommandés actuellement par l’OMS pour le traitement de la TBP-MR/RR [15]. La majorité (83,9 %) des 322 patients atteints de TBP-RR inclus dans cette étude présentait une TBP-MR. Nos résultats confirment clairement que dans notre milieu, la résistance à la R peut servir de marqueur de substitution pour la TBP-MR. Cependant, il convient de noter que cette avancée majeure qui utilise le test Xpert MTB/ RIF seul pour identifier les patients à traiter par le régime la TBP-MR de 9 à 11 mois, peut contribuer à des pratiques de soins sous-optimales, car leur traitement n’est pas guidé par des résultats de tests de sensibilité pour tous les médicaments inclus dans ce régime thérapeutique. Dans cette étude, 11 (3,9 %) des 281 patients atteints de TBP-RR résistants à au moins un médicament testé présentaient une tuberculose pré-ultrarésistante aux médicaments et 3 (1,1 %) avaient en outre une résistance à un aminoside. Cette découverte de notre étude a plusieurs implications importantes : premièrement, il existe un risque élevé que le régime standardisé de 9 à 11 mois actuellement en vigueur pour le traitement des patients atteints de TBP-MR/RR au Cameroun soit inadéquat, car il pourrait ne pas contenir, comme le recommande l’OMS, au moins quatre médicaments susceptibles d’être efficaces lorsqu’ils sont prescrits, par exemple, à un patient atteint de TBP pré-UR [16]; deuxièmement, si le régime de traitement est sous-optimal, les chances de guérison peuvent être réduites et une amplification de la résistance aux médicaments peut se produire, de sorte qu’une souche de TBP pré-UR peut devenir une souche de TBP-UR. Cela peut à son tour être transmis à d’autres personnes au sein de la famille du malade et de la communauté.

Il est donc nécessaire que tous les patients à risque d’avoir une tuberculose active due à une souche résistante aux médicaments, bénéficient d’un test rapide de sensibilité à la R et, en cas de résistance à la R, de sensibilité à la H, l’E, fluoroquinolones, et autres médicaments de deuxième ligne lors de l’évaluation initiale. Cela permettra un ajustement rapide de leur traitement.

Bien entendu, cela nécessitera un investissement plus important pour le renforcement des capacités de laboratoire afin de répondre aux besoins des patients tuberculeux et des cliniciens. Au cas où ce renforcement se révélerait impossible, il faudrait que le PNLT remplace rapidement le régime actuel de 9 à 11 mois pour le traitement de la TBP-MR/ RR par le nouveau régime court recommandé par l’OMS [15]. Ce nouveau régime est composé des nouveaux médicaments antituberculeux tels que la Bdq, le prétomanide et le linézolide avec ou sans Mfx auxquels les patients atteints de TBP-MR/RR n’ont pas encore pour la plupart été antérieurement exposés.

Notre étude peut avoir certaines limites. Premièrement, pendant la période qu’elle couvre, la définition de la TB-UR recommandée par l’OMS a changé pour devenir « la tuberculose causée par des souches de *M. tuberculosis* qui répondent à la définition de la TBP-MR/RR et sont également résistantes à toute fluoroquinolone et à au moins un médicament supplémentaire du groupe A » [15]. Auparavant, ce médicament supplémentaire du groupe A était un aminoside qui a été remplacé. Cependant, ce changement de définition n’a eu aucune conséquence significative sur les résultats de notre étude et la discussion de ceux-ci. Deuxièmement, seuls les patients atteints de TBP-RR admis consécutivement au centre spécialisé de traitement de la TBP-MR de l’HJY au cours de la période d’étude ont été inclus. Par conséquent, il peut y avoir un certain biais quant à la représentativité de notre population d’étude par rapport à l’ensemble de la population des malades atteints de TBP-RR à Yaoundé et de ses environs. Néanmoins, le centre spécialisé de traitement de la TBP-MR du HJY est le seul établissement de référence et de traitement qui prend en charge ce groupe de patients depuis plus d’une décennie à Yaoundé et ses environs. La série de malades de cette étude peut donc être considérée comme un reflet adéquat des patients atteints de TBP-RR dans cette zone.

Enfin, notre travail peut également avoir les limitations habituelles inhérentes à une conception rétrospective de l’étude. Nous pensons cependant que la nature rétrospective de l’étude n’aura qu’un impact minimal sur nos résultats en raison de la robustesse des activités de supervision et de suivi régulières du centre spécialisé de traitement de la TBP-MR par le PNLT. Malgré ces limitations, notre étude fournit des données de base pertinentes et utiles sur la situation de la résistance aux médicaments antituberculeux chez les patients atteints de TBP-RR à Yaoundé, au Cameroun.

## Conclusion

Le taux global de résistance aux antituberculeux des souches du complexe *M. tuberculosis* chez les patients atteints de TBP-RR à Yaoundé est élevé. Cette résistance concerne le plus souvent deux médicaments, à savoir la H et l’E. Dans leur majorité, les patients atteints de TBP-RR ont une tuberculose multirésistante. Le taux de la TBP pré-UR chez ceux qui sont atteints de TBP-RR est relativement faible.

Cette étude souligne que la stratégie de détection des cas de résistance aux médicaments basée sur un test rapide qui identifie uniquement la résistance à la R comme marqueur de substitution de la TBP-MR, peut conduire à l’instauration d’un traitement sous-optimal pour un nombre non négligeable de patients traités par le régime de 9 à 11 mois. Cela peut à son tour contribuer à l’amplification de la résistance et à la transmission de souches présentant une résistance de plus en plus avancée au sein de la communauté.

Ainsi, nous recommandons que le PNLT du Cameroun adopte et généralise rapidement le nouveau régime thérapeutique recommandé par l’OMS, compte tenu de la non-disponibilité dans le pays des tests rapides de sensibilité permettant de détecter la résistance ou non aux médicaments utilisés dans le régime de 9 à 11 mois.

## Contributions des auteurs

AK et CK ont conceptualisé l’étude et ont collecté et analysé les données; CK a rédigé le manuscrit; CK et JN ont révisé la version finale du manuscrit. Tous les auteurs ont eu accès à l’ensemble des données de l’étude, assument la responsabilité de l’exactitude de l’analyse et ont eu autorité sur la préparation du manuscrit et la décision de soumettre le manuscrit pour publication. Tous les auteurs ont approuvé la version finale et acceptent sa soumission.

## Disponibilité des données

Toutes les données sont disponibles sur demande auprès d’AK.

## Sources de financement

Cette recherche n’a reçu aucune forme de subvention d’un organisme de financement, qu’il soit gouvernemental, commercial ou à but non lucratif.

## Déclaration de liens d’intérêts

Les auteurs déclarent n’avoir aucune relation financière ou personnelle qui aurait pu les influencer de manière inappropriée dans la rédaction de cet article.
