# Supplementary material for: Patterns of resistance to anti-tuberculosis drugs among patients with rifampicin-resistant pulmonary tuberculosis in Yaoundé, Cameroon
Source: Med Trop Sante Int. 2026 Jun 15;6(2):mtsi.v6i2.2026.865. [Article in French] doi: 10.48327/mtsi.v6i2.2026.865 (PMC13423294; doi:10.48327/mtsi.v6i2.2026.865)
Supplement: Related texts

# Research article: Patterns of resistance to anti-tuberculosis drugs among patients with rifampicin-resistant pulmonary tuberculosis in Yaoundé, Cameroon

## Introduction

Pulmonary tuberculosis (PTB) is the leading cause of death from a single infectious agent worldwide, surpassing the human immunodeficiency virus infection/acquired immunodeficiency syndrome (HIV/AIDS) [13].

Despite the considerable efforts deployed, its control is in part hampered by drug-resistant PTB, particularly multidrug-resistant (MDR-PTB). This form is defined as PTB that is resistant to at least rifampicin (R) and isoniazid (H). These two drugs are the most effective, least expensive, and best tolerated for treating drug-susceptible PTB [15]. However, it has been demonstrated that the vast majority of patients with strains of R-resistant PTB (RR-PTB) have a similar poor prognosis as MDR-PTB cases when treated solely with first-line anti-TB drugs [4,8]. For this reason, patients with RR-PTB are now treated as MDR-PTB cases. The availability of the Xpert MTB/RIF test (Cepheid, Sunnyvale, CA) in Cameroon, which not only detects *Mycobacterium tuberculosis* in specimens but also its resistance to rifampicin, allows for the rapid screening of suspected cases of rifampicin-resistant PTB and the immediate treatment with the MDR-PTB regimen if indicated. This regimen comprises first- and second-line anti-TB drugs.

While this can be considered a significant step forward, it has to be acknowledged that the use of the Xpert MTB/RIF test alone to identify patients to be treated with the 9-11-month standardized MDR-PTB treatment could result in suboptimal treatment practices. This may be due to the fact that the treatment is not entirely guided by laboratory-confirmed drug susceptibility test (DST) results for all included drugs. The absence of DST results for these patients at the start of treatment may lead to unidentified drug resistance to both first- and second-line drugs. This is even more true nowadays given that resistance to both new and reused drugs is now reported to be emerging [9,10–12].

Knowledge of the patterns of drug resistance in RR-PTB patients is therefore essential in our setting to guide management and reduce the risk of further resistance. This study analyzed the patterns of drug resistance to anti-tuberculosis drugs observed in RR-PTB patients who received the standardized 9-11-month treatment regimen for MDR-PTB in Yaounde, Cameroon.

## Materials and methods

This study was conducted at the specialized MDR-PTB treatment center of the Jamot Hospital in Yaounde (JHY), the sole referral facility for Yaounde and the surrounding area.

In this center and in accordance with the guidelines of the National Tuberculosis Control Program (NTCP) of Cameroon [5] and the recommendations of the World Health Organization (WHO) [15], all patients with previously treated pulmonary tuberculosis (PTB), as well as contacts of multidrug-resistant/rifampicin resistant PTB (MDR/RR-PTB) patients presenting with symptoms suggestive of PTB, submit two sputum specimens on two consecutive days for microscopic examination in search of acid fast bacilli at the National Tuberculosis Reference Laboratory at the Centre Pasteur du Cameroun in Yaoundé. The specimens are also tested using the Xpert MTB/RIF test to determine if they contain *M. tuberculosis* strains that are resistant or not to rifampicin. Patients’ results are immediately communicated to the specialized center for appropriate therapeutic management. The remaining sputum specimens received at the reference laboratory from patients with rifampicin resistance on the Xpert MTB/RIF test are cultured in liquid media. Culture and drug susceptibility testing for antituberculosis drugs in liquid media were performed using BD BACTEC MGIT 960 (BD Diagnostics; Sparks, USA). BACTEC MGIT 7ml tubes with the MGIT supplementation kit, and the combined PANTA and OADC vials were used for culture, and the BACTEC MGIT SIRE kit was used for first-line antituberculosis drug susceptibility testing.

A DST for first-line and certain second-line drugs on M. tuberculosis strains isolated from cultures was performed using the MTBDR plus Genotype Version 2 Strip Hybridization Assay (Bruker, Nehren, Germany) and one of the conventional phenotypic drug susceptibility test using the indirect proportion method described by Canetti et al. [6] and/or the Mycobacteria Growth Indicator Tube (BD BACTEC MGIT 960). The WHO critical concentrations recommended in Module 3 of its Operational Manual on Tuberculosis [16] were used for the antituberculosis drug susceptibility tests.

The drugs currently tested in the reference laboratory include the following first-line drugs: R, H, and ethambutol (E), as well as second-line drugs: quinolones (ofloxacin and moxifloxacin) and aminoglycosides (amikacin and kanamycin). Culture and DST results are reported to the specialized treatment center, where they are recorded in the MDR-PTB treatment register and on each patient’s treatment record.

Up until 2022, all patients with MDR/RR-PTB received a standardized 9-11-month treatment. This regimen included a 4-6-month intensive phase with the daily administration of the following drugs: Moxifloxacin (Mfx), amikacin (Am), prothionamide (Pto), clofazimine (Cfz), high-dose H (Hh), E, and pyrazinamide (Z) [4-6Am-Mfx-Pto-Cfz-Hh-E-Z]. This phase was followed by an additional five-month phase comprising Mfx, Cfz, E, and Z, also administered daily. In 2023, the regimen was modified by replacing Am with bedaquiline (Bdq), administered during the first six months of treatment (4–6 Bdq–Mfx–Cfz–Z–E–Hh–Pto/5 Mfx–Cfz–Z–E).

This was a retrospective cross-sectional study, including all patients aged 15 years and older with RR-PTB who were put on treatment at the specialized MDR-PTB treatment center of the JHY from January 2013 to December 2022. Patients with incomplete records were excluded from the study. Those whose Xpert MTB/RIF tests showed a rifampicin-resistant strain of M. tuberculosis but whose cultures were contaminated or showed no growth were also excluded.

All eligible RR-PTB patients who initiated treatment during the study period were identified using the MDR-PTB treatment register and their treatment records. For each patient, the following information was extracted from these documents for each patient and recorded on a pre-prepared data collection form: registration number, age, sex, patient category based on prior anti-TB treatment history (relapse, treatment failure, retreatment failure, return after loss to follow-up, or contact with a known case of MDR/RR-PTB), and HIV status.

The results of the drug susceptibility test for each patient included were also extracted and recorded.

The pattern of resistance of these patients were further categorized according to the following definitions [15]:

- Patient with MDR-PTB: defined as a patient whose sputum culture isolate is positive for *M. tuberculosis* and whose in vitro resistance to at least H and R is established.
- A patient with pre-extensively drug-resistant tuberculosis (pre-XDR-PTB): defined as a patient with a MDR-PTB strain that is resistant to fluoroquinolones (ofloxacin or Mfx).
- Patient with extensively drug-resistant tuberculosis (XDR-PTB): defined as a patient harbouring strains that meet the definition of MDR/RR-PTB and are also resistant to fluoroquinolones and at least one additional Group A drug.

Data were double-entered into a computer using EpiData Entry Client software (version 4.6.0.6). Discordances were identified and resolved by verification of the original paper records. Data analysis was performed using the EpiData Analysis software, version 3.3.0. Descriptive analyses were done for demographic, clinical, and laboratory variables and presented as means with standard deviations (SD), counts, and/or percentages.

The study was approved by the Institutional Review Board (IRB) of the Faculty of Medicine and Biomedical Sciences of The University of Yaounde 1 (reference no. 1264/UY1/FMSB/ VDRC/DAASR/ CSD). As this was a retrospective study, informed consent from participants could not be obtained. Patient information was anonymized and depersonalized prior to analysis. Administrative authorization to carry out the study at the Specialized Treatment Centre for multidrug-resistant tuberculosis was obtained for the administration of the (Ref. No.: 444/L/ MINSANTE/SG/DHJY/MM). The study was carried out in accordance with the 2013 revision of the Declaration of Helsinki.

## Results

A total of 351 RR-PTB patients were enrolled on treatment at the Specialized center for MDR-PTB of the JHY during the study period. Of these patients, 29 (8.3%) were excluded due to incomplete records, including the absence of drug susceptibility test results caused by the absence of M. tuberculosis growth in culture or contaminated cultures. However, no statistically significant differences were observed in terms of age, sex, or HIV status between the excluded patients and the 322 patients included in the study.

Of these 322 patients, 198 (61.5%) were male and 124 (38.5%) were female, with a mean age of 36.2 ± 12.7 years (range 15–81 years). One hundred and one of them (31.4%) were HIV-positive, and the majority (258 patients, 80.1%) had previously been treated for tuberculosis. Table I summarizes the general characteristics of the 322 patients included in the study.

**Table I:** General characteristics of the study population

Characteristics	Number (N = 322)	%
Age (in years)		
mean ± standard deviation	36,2 ± 12,7	
range	15-81	
Gender		
male	198	61,5
female	124	38,5
HIV status		
positive	101	31,4
negative	221	68,6
Category of previous PTB treatment		
failure of first-line treatment (FLT)	121	37,6
relapse after FLT for new cases	110	34,2
return after discontinuation of FLT for new cases	27	8,4
new PTB case in contact with a known case of MDR/RR-PTB	49	15,2
other	15	4,7

Among the 322 RR-PTB patients studied, 41 (12.7%) were susceptible to all of the antituberculosis drugs that were tested, except for R. Resistance to one or more drugs was observed in 281 patients (87.3%). All the 281 RR-PTB patients with additional resistance were found to be resistant to H, followed by E in 136 (48.4%) of these patients. Resistance to H alone was observed in 139 (43.2%) of the 322 RR-PTB patients included and was associated with resistance to the other tested drugs in the remaining 183 cases (56.8%). The patterns of resistance to the tested antituberculosis drugs are presented in Table II.

**Table II:** Resistance profiles to other antituberculosis drugs among 281 RR-PTB patients in Yaoundé, Cameroon

Resistant to the drug(s)	Study population (n)	% of the resistant population (n = 281)
One medication		
H	139	43,2
Two medications		
HE	128	39,6
HFq	5	1,6
HKm	1	0,3
Total	134	41,6
Three medications		
HEFq	3	0,9
HEKm	2	0,6
Total	5	1,6
Four medications		
HEKmFq	3	0,9
At least one medication	281	87,3

H = isoniazide; E = ethambutol; Fq = fluoroquinolones; Km = kanamycine

Of the 281 RR-PTB patients with resistance to at least one drug, 139 (49.6%) were resistant to a single drug, 134 (47.6%) were resistant to two drugs, and eight (2.9%) were resistant to three or more drugs. Resistance to H was always present when resistance to another drug was present.

Table III presents the distribution of the 322 RRPTB patients who were resistant to at least one of the tested drugs according to their class of drug resistance pattern.

**Table III:** Distribution of 322 RR-PTB patients by class of antituberculosis drug resistance profiles

Drug resistance profile	RR-PTB patients (n = 322)	%
Resistance to R only	41	12,7
Multidrug resistance	270	83,9
Pre-extreme drug resistance	11	3,4
**Total**	**322**	**100,0**

The proportion of patients with solely MDR-PTB strains was 83.9%, whereas the proportion with pre-XDR-PTB was only 3.4%.

## Discussion

To our knowledge, this is the first study to examine resistance profiles to first- and second-line antituberculosis drugs in patients with drug-resistant tuberculosis in Cameroon.

The overall rate of resistance to at least one of the tested anti-tuberculosis drugs among these patients was 87.3%. This rate is consistent with similar results observed in Sierra Leone and Uganda [1,2]. The overall high rate of drug resistance can be explained by the fact that most RR-PTB patients included in our study (84.5%) had a history of prior anti-tuberculosis treatment. Forty-one cases were resistant to R alone. Monoresistance to R is quite rare and these cases require further analysis of the patients’ clinical records.

Most patients with RR-PTB (281 patients, or 87.3%) were resistant to at least one of the tested drugs, with H being the most frequently drug concerned, followed by E (48.4%). The high rates of resistance to H and E in RR-PTB patients call into question the appropriateness of including them in the 9-11-month all-oral regimen for people with MDR/RR-PTB, a regimen still used in Cameroon for a large proportion of these patients. It is now established that mutations in the *inhA* promoter or in the *inhA* and *KatG* regions of *M. tuberculosis* confer resistance to H. The resistance is considered low-level when only *inhA* mutations are present and high-level when mutations are observed in either the *KatG* gene alone or in combination with mutations in the *inhA* promoter and *KatG* regions. It is also known that mutations in the *inhA* promoter are associated with resistance to ethionamide and prothionamide. Prothionamide is included in the 9-month all-oral regimen. Currently, these mutations cannot be detected in Cameroon. Consequently, the presence of mutations of the *inhA* promoter and of *KatG* suggests that high-dose H and thionamides may be ineffective and that the 9-11-month all-oral regimen may be unsuitable for all patients with MDR/RR-PTB [15]. However, it has been proposed that, in the absence of information on mutation patterns for a given patient in countries such as Cameroon, the decision to treat a patient with this regimen can be informed by the frequency with which both mutations occur concurrently, as obtained from drug resistance surveillance [14]. Unfortunately, like many low- and middle-income countries, our country does not currently have surveillance systems in place to provide information on these mutations.

Resistance to the two second-line drugs tested in this study was low. Resistance to fluoroquinolones was observed in 11 (3.9%) of the 281 RR-PTB patients who were resistant to at least one of the drugs tested. This rate is lower than the 7% rate reported by Rao et al. in Pakistan [17]. Since no data are currently available regarding RR-PTB susceptibility to quinolones in Cameroon, this finding is significant. The rate of quinolone resistance observed in this study, though low, should be considered a warning sign for treating this group of patients with tuberculosis, as it could lead to cases of extensive drug resistance. The current situation could eventually worsen because quinolones can be used in Cameroon both on a self-administered basis and by prescription for the treatment of infectious diseases other than tuberculosis. It is of paramount importance to issue warnings against the misuse of this major second-line drug, as higher rates of quinolone resistance would threaten the efficacy of the shorter, fully oral regimens currently recommended by the WHO for treating MDR/RR-PTB [15].

The majority (83.9%) of the 322 RR-PTB patients included in this study had MDR-PTB. Our results clearly confirm that, in our setting, resistance to R can serve as a surrogate marker for MDR-PTB. However, this major advancement which uses the Xpert MTB/RIF test alone to identify patients to be treated with the 9-11-month MDR-PTB regimen, may contribute to suboptimal care practices because their treatment is not guided by susceptibility test results for all drugs included in the regimen. In this study, 11 (3.9%) of the 281 RR-PTB patients resistant to at least one tested drug had pre-extensively resistant TB, and three (1.1%) had additional resistance to an aminoglycoside. This finding from our study has several important implications:

Firstly, there is a high risk that the currently prevailing standardized 9-11-month regimen for the treatment of multidrug-resistant/rifampicin-resistant tuberculosis (MDR/RR-PTB) patients in Cameroon is inadequate as It may not contain as recommended by the WHO, at least four drugs that are likely to be effective when prescribed for example to a patient with pre-extensively drug-resistant tuberculosis (pre-XDR-PTB) [16]. Secondly, if the treatment regimen is suboptimal, the chances of a cure may be reduced, and an amplification of drug resistance may occur such that a pre-XDR-PTB strain becomes XDR-PTB strain. This can, in turn, be transmitted to other persons within the patient’s family and the community. It is therefore necessary for all patients at risk of having active tuberculosis caused by a drug-resistant strain to benefit from a rapid drug-susceptiblity test for resistance to R and, in the event of resistance to R, testing for sensitivity to H, E, fluoroquinolones, and other second-line drugs during the initial evaluation. This will allow for a rapid adjustment of treatment. Of course, this will require greater investment in reinforcing laboratory capacities to meet the needs of TB patients and clinicians. In a situation where this reinforcement is impossible, the NTCP would need to rapidly replace the current 9-11-month regimen for treating MDR/RR-PTB with the new, shorter regimen recommended by the WHO [15]. This new regimen made up of new anti-tuberculosis drugs, such as bedaquiline (Bdq), pretomanid, and linezolid, with or without moxifloxacin (Mfx), to which most patients with MDR/RR-PTB have not been previously exposed. Our study may have certain limitations. Firstly, during the period covered by the study the WHO recommended definition of XDR-TB changed to become “tuberculosis caused by strains of M. tuberculosis that meet the definition of MDR/RR-PTB and are also resistant to any fluoroquinolone and at least one additional Group A drug”[15]. Previously, the additional Group A drug was an aminoglycoside which has been replaced. However, this change in the definition had no significant impact on our study’s results and their discussion. Secondly, only patients with RR-PTB admitted consecutively into the specialised MDR-TB treatment centre of the JHY over the study period were included. Consequently, there may be some bias with regard to our study population being representative of the RR-PTB patients in Yaoundé and its environs. Nevertheless, the specialised MDR-TB treatment center of the JHY is the sole referral and treatment facility that has been caring for this group of patients for over a decade in Yaoundé and its surrounding areas. The patient series in this study can therefore be considered an adequate reflection of RR-PTB patients in this area .

Finally, our study may also have the usual limitations inherent to a retrospective study design. We, however, believe that the retrospective nature of the study will only have a minimal impact on our results due to the robustness of the regular supervision and monitoring activities of the specialised MDR-TB treatment center by the NTCP. Despite these limitations, our study provides relevant and useful baseline data on antituberculosis drug resistance in patients with RR-PTB in Yaoundé, Cameroon.

## Conclusion

The overall rate of drug resistance among *M. tuberculosis* complex strains in RR-PTB patients in Yaounde is high. This resistance most often involves two drugs, namely H and E. The majority of RR-PTB patients have multidrug-resistant tuberculosis. The rate of pre-XDR-PTB among RR-PTB patients is relatively low.

This study shows that using a rapid test to detect only resistance to R as a surrogate marker for MDR-PTB may lead to suboptimal treatment for a significant number of patients treated with the 9-11-month regimen. This may contribute to the amplification of resistance and the transmission of increasingly resistant strains within the community.

Therefore, we recommend that the NTCP of Cameroon should rapidly adopt and roll out the new treatment regimen recommended by the WHO, due to the unavailability of rapid susceptibility tests in the country that can detect resistance to the drugs used in the 9-11-month regimen.

## Authors’ contributions

AK and CK designed the study, collected and analyzed the data; CK drafted the manuscript; CK and JN reviewed the final version of the manuscript. All authors had access to all study data, assume responsibility for the accuracy of the analysis, and had authority over the preparation of the manuscript and the decision to submit the manuscript for publication. All authors approved the final version and agree to its submission.

## Data Availability

All data are available upon request from AK.

## Funding

This research did not receive any grant funding from governmental, commercial, or nonprofit sources.

## Conflicts of interest

The authors declare that they have no financial or personal relationships that could have inappropriately influenced them in writing this article.
